# Mechanism of immune escape mediated by receptor tyrosine kinase KIT in thyroid cancer

**DOI:** 10.1002/iid3.851

**Published:** 2023-07-12

**Authors:** Zhen Luo, Jin Xu, Dayong Xu, Jiaojiao Xu, Rongjun Zhou, Keping Deng, Zheng Chen, Fang Zou, Libo Yao, Yuqin Hu

**Affiliations:** ^1^ Department of General Surgery Minimally Invasive Surgery Center, The First Hospital of Changsha Changsha Hunan China; ^2^ Department of Surgery Changsha Hospital for Maternal and Child Health Care Changsha Hunan China

**Keywords:** CD8+ T cells, immune escape, KIT, MAPK pathway, migration and invasion, programmed death‐ligand 1, thyroid cancer

## Abstract

**Objective:**

Thyroid cancer (TC) is one of the fastest‐growing malignant tumors. This study sought to explore the mechanism of immune escape mediated by receptor tyrosine kinase (KIT) in TC.

**Methods:**

The expression microarray of TC was acquired through the GEO database, and the difference analysis and Kyoto encyclopedia of genes and genomes pathway enrichment analysis were carried out. KIT levels in TC cell lines (K1/SW579/BCPAP) and human normal thyroid cells were detected using reverse transcription quantitative polymerase chain reaction and western blot analysis. TC cells were transfected with overexpressed (oe)‐KIT and CD8+ T cells were cocultured with SW579 cells. Subsequently, cell proliferation, migration, and invasion abilities, CD8+ T cell proliferation, cytokine levels (interferon‐γ [IFN‐γ]/tumor necrosis factor‐α [TNF‐α]) were determined using colony formation assay, Transwell assays, flow cytometry, and enzyme‐linked immunosorbent assay. The phosphorylation of MAPK pathway‐related protein (ERK) was measured by western blot analysis. After transfection with oe‐KIT, cells were treated with anisomycin (an activator of the MAPK pathway), and the protein levels of p‐ERK/ERK and programmed death‐ligand 1 (PD‐L1) were detected.

**Results:**

Differentially expressed genes (*N* = 2472) were obtained from the GEO database. KIT was reduced in TC samples and lower in tumor cells than those in normal cells. Overexpression of KIT inhibited immune escape of TC cells. Specifically, the proliferation, migration, and invasion abilities of TC cells were lowered, the proliferation level of CD8+ T cells was elevated, and IFN‐γ and TNF‐α levels were increased. KIT inhibited the activation of the MAPK pathway in TC cells and downregulated PD‐L1.

**Conclusion:**

KIT suppressed immune escape of TC by blocking the activation of the MAPK pathway and downregulating PD‐L1.

## Graphical abstract

KIT suppressed immune escape of thyroid cancer by blocking the activation of the MAPK pathway and downregulating programmed death‐ligand 1.

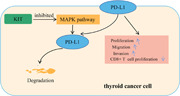

## 1 INTRODUCTION

Thyroid cancer (TC) is a malignant tumor originating from thyroid follicular cells or parafollicular cells.^1^ In recent years, TC, as the most common endocrine malignant tumor, has had an increasing incidence.^2^, ^3^ There are many types of TC, papillary thyroid carcinoma (PTC) is the most frequent in clinical practice, accounting for about 80%.^4^ Although the vast majority of PTC patients can be treated with surgery, radioactive iodine, and thyroxine suppression, 10% of patients die from poorly differentiated and advanced tumors.^5^ Part of the reason is that during the formation of tumors, they are closely related to the immune microenvironment around tumor cells, which collectively promote the immune function tolerance of the body and then develop into the immune escape, leading to the body's inability to clear tumors.^6^ Consequently, understanding the immune escape mechanism of TC is very crucial for the treatment of TC.

The immune system interacts closely with tumor cells in the host body to inhibit or promote the occurrence of tumors.^7^, ^8^ Tumor cells can develop a variety of immune escape mechanisms to manipulate the host immune system and immune microenvironment to avoid recognition and elimination by immune cells.^3^ The main methods include upregulation of immunosuppressive molecules, downregulation of antigens, and recruitment of inhibitory cell populations.^9^ Programmed death‐ligand 1 (PD‐L1), a programmed T cell death receptor protein, is also an important immunosuppressant molecule in the human body, and it can bind with its ligand PD‐L1 to inhibit the activation of antigen‐specific CD8+ T cells, thus promoting the immune escape of tumor cells.^10^ A study has found that PD‐1 is highly expressed in a variety of tumor cells, and recently it has been used as a cancer immunotherapy target.^11^ In addition, its ligand PD‐L1 is also abnormally high expressed in tumor cells, which is also a major factor promoting tumor immune escape ability.^12^ The expression of PD‐L1/PD‐1 in various cancers has been studied, and blocking the PD‐L1/PD‐1 pathway is the cornerstone of immunotherapy.^13^ Therefore, it is important to understand the specific mechanism of PD‐L1 in immune escape in TC.

Receptor tyrosine kinase (RTK), as a cell surface receptor involved in mediating intercellular communication, controls multiple signaling, and biological functions, and their imbalance can lead to abnormal activation of downstream intracellular signaling pathways, resulting in many human diseases, such as diabetes, inflammation, and cancer.^14^ The KIT gene, also known as c‐KIT or CD117, encodes RTK III activated by stem cell factor.^15^, ^16^ It has been reported that c‐KIT/CD117 expression is downregulated in PTC and can be used as a new auxiliary marker of PTC.^17^ Meanwhile, c‐KIT has also been reported to be deficient in TC cells and involved in tumor differentiation and development.^18^ KIT acts on a targeted pathway that coordinates the number and activity of tumor innate immune cells.^19^ It was reported that KIT is involved in signal transduction by governing different downstream pathways such as MAPK, PI3K, and JAK/STAT.^20^ Nevertheless, whether KIT mediates immune escape of TC by coordinating the expression of PD‐L1 through the MAPK pathway has not been reported. The purpose of this study was to explore the mechanism of KIT‐mediated immune escape in TC and provide a new therapeutic target for TC.

## 2 MATERIALS AND METHODS

### 2.1 Bioinformatics analysis

TC expression microarray was obtained from the GEO database (https://www.ncbi.nlm.nih.gov/geo/). Using |logFC| >1 and *p* < .05 as the screening criteria, “limma” package of R language was used for differential analysis to identify differentially expressed genes (DEGs). The “clusterProfile” package was used for Kyoto encyclopedia of genes and genomes (KEGG) pathway enrichment analysis of DEGs. KEGG database (https://www.kegg.jp/kegg/pathway.html) was adopted to search the location of candidate gene KIT in the MAPK pathway. Differential expression of KIT in TC and normal samples collected by The Cancer Genome Atlas (TCGA) and Genotype‐Tissue Expression (GTEx) was analyzed using the GEPIA2 database (http://gepia2.cancer-pku.cn/#index).

### 2.2 Cell culture and treatment

TC cell lines (K1, SW579, and BCPAP) and human normal thyroid cell lines (HT‐ori3) were purchased from The Cell Bank of Type Culture Collection of The Chinese Academy of Sciences. K1 and BCPAP cells were cultured in RPMI‐1640 medium (Invitrogen) containing 10% fetal bovine serum (FBS; Thermo Fisher Scientific) and 1% P/S (100 U/mL penicillin and 100 μg/mL streptomycin) (Thermo Fisher Scientific) in an incubator at 37°C with 5% CO_2_. SW579 cells were cultured in L‐15 medium (Invitrogen) containing 10% FBS and 1% P/S and stored in an incubator with 5% CO_2_ at 37°C. All cell lines were verified and tested by the short Tandem repeat method.

HT‐ori3 cells were cultured in Dulbecco's modified Eagle medium containing 10% FBS and 1% P/S in an incubator with 5% CO_2_ at 37°C.

### 2.3 Cell transfection and grouping

SW579 cells were allocated into the following five groups: blank group (without any treatment), overexpressed (oe)‐negative control (NC) group (cells transfected with oe‐NC were cultured for 24 h), oe‐KIT group (cells transfected with oe‐KIT were cultured for 24 h), oe‐KIT + dimethyl sulfoxide (DMSO) group (cells transfected with oe‐KIT were cultured for 24 h and then treated with DMSO for 1 h), and oe‐KIT + Anisomycin (cells transfected with oe‐KIT were cultured for 24 h, then treated with the MAPK pathway activator Anisomycin [0.1 μmol/L] for 1 h).^21^ Anisomycin was purchased from Yuanye Biotech Co., Ltd. oe‐KIT and its NC plasmids all purchased from GenePharma were transfected into SW579 cells using Lipofectamine 2000 (Invitrogen) at a final concentration of 50 nM.^22^

### 2.4 Coculture of CD8+ T cells and SW579 cells

CD8+ T cells were purchased from Liver Biotech Co., Ltd. CD8+ T cells and SW579 cells were cocultured in a double chamber coculture system (Millipore). CD8+ T cells were stimulated with monoclonal antibodies anti‐CD3 and anti‐CD28 before coculture. SW579 cells were seeded in the upper transwell chamber and CD8+ T cells were seeded in the lower chamber. The cells were cocultured in RPMI‐1640 medium containing 10% FBS for a certain period.

### 2.5 Reverse transcription quantitative polymerase chain reaction (RT‐qPCR)

Total RNA was extracted from TC cells using the TRIzol reagent (Invitrogen) and transcribed into cDNA using the PrimeScript RT kit (Takara), and the concentration and purity of RNA samples were determined by the spectrometric method. qPCR was performed using SYBR® Premix Ex TaqTM II (Takara) on ABI7900HT fast PCR real‐time system (ABI). Reaction conditions were as follows: pre‐denaturation at 94°C for 10 min, with 40 cycles of denaturation at 94°C for 45 s, annealing at 60°C for 45 s, and extension at 72°C for 45 s. Using glyceraldehyde‐3‐phosphate dehydrogenase (GAPDH) as an internal reference gene, the relative expression of KIT mRNA standardized by internal reference was calculated by the 2^−ΔΔCt^ method. The primers were synthesized by Sangon Biotech. The sequences are shown in Table 1.^18^

**Table 1:** Primer sequences.

Primer	Sequence
KIT	Forward: 5′‐GCACCTGCTGCTGAAATGTATGACATAAT‐3′
	Reverse: 5′‐TTTGCTAAGTTGGAGTAAATATGATTGG‐3′
GAPDH	Forward: 5′‐GCACCGTCAAGGCTGAGAAC‐3′
	Reverse: 5′‐ATGGTGGTGAAGACGCCAGT‐3′

### 2.6 Colony formation experiment

SW579 cells were seeded in 12‐well plates at 1 × 10^4^ cells/well. Once the cell density reached 80%, oe‐KIT was transfected into SW579 cells by Lipofectamine 2000 (Invitrogen). Then the cells were seeded in six‐well plates (200 cells/well) and cultured for 8 days. Afterwards, the cells were fixed with formalin for 30 min and then stained with Giemsa (Sigma‐Aldrich) to count colonies with more than 50 cells.

### 2.7 Transwell assay

Transwell chamber (Thermo Fisher Scientific; 140644) was placed into a 24‐well plate, and 500 mL medium containing 20% FBS was added to the lower chamber. Then the 24‐well plate was placed in a cell incubator for 2 h. Cells in the logarithmic growth stage were detached with trypsin and washed with phosphate buffer saline (PBS) or serum‐free medium three times. The cells were resuspended and counted, and the cell concentration was adjusted to 2 × 10^5^/mL. After the cells were mixed, 500 mL suspension was added to the upper chamber and no bubbles were allowed between the upper and lower chambers. Matrigel was added to the upper chamber to test cell invasion ability. In the migration experiment, the upper chamber of Transwell did not contain Matrigel. The 24‐well plate was placed in the cell incubator for further culture. After 20 h, the fluid in the upper chamber was sucked and cells were washed once with PBS and then moved to the well containing 800 mL methanol or paraformaldehyde and fixed at room temperature for 30 min. Then the chamber was cleaned twice with PBS and stained with 800 μL crystal violet at room temperature for 15−30 min in a condition devoid of light. After staining, the chamber was gently washed and soaked several times with double distilled water until the color of the staining solution was washed away. The liquid in the upper chamber was sucked away, and the cells on the membrane surface at the bottom of the upper chamber were carefully wiped with a wet cotton swab. Then 10 fields were randomly selected and photographed under a microscope (×20). The number of cells in each photo was calculated and the average was applied for statistical analysis.

### 2.8 Proliferation test of CD8+ T cells

CD8+ T cells labeled with carboxyfluorescein succinimidyl ester (CFSE) (S1076; Solarbio) were cultured with SW579 cells in the RPMI‐1640 medium with 5% CO_2_ at 37°C for 5 days. CFSE levels were detected by flow cytometry.

### 2.9 Western blot analysis (WB)

SW579 cells were lysed with radioimmunoprecipitation assay (Beyotime) lysate, and the protein concentration was determined using the bicinchoninic acid protein detection kits (Beyotime). The 10% sodium dodecyl sulfate‐polyacrylamide (SDS‐PAGE) gel was prepared, and then the protein was separated in SDS‐PAGE gel and electrotransferred to polyvinylidene fluoride (PVDF) membranes (Millipore). Tris‐buffered saline tween (TBST) was used to prepare 5% skim milk, and then PVDF membranes were put into the milk, shaken, and blocked at room temperature for 1 h to block the nonspecific binding. The membranes were added with primary anti‐KIT (ab283653; 1:1000; Abcam), anti‐p‐extracellular signal‐regulated kinases (ERK) (ab229912; 1:1000; Abcam), anti‐ERK (ab32537; 1:1000; Abcam), anti‐PD‐L1 (ab205921; 1:100; Abcam), anti‐GAPDH (ab8245; 1:5000; Abcam), and incubated overnight at 4°C. The membranes were washed with TBST twice and incubated with horseradish peroxidase‐labeled goat anti‐rabbit IgG (ab48386; 1:2000; Abcam) at room temperature for 1 h. Next, the membranes were developed with enhanced chemiluminescence working fluid (Millipore) and photographed. ImageJ software (version 1.48; NIH) was adopted to detect protein band density, with GAPDH as an internal reference.

### 2.10 Enzyme‐linked immunosorbent assay (ELISA)

TC cells were lysed with RIPA lysate containing protease inhibitors, and the supernatant was collected by centrifugation (14,000 × *g*, 5 min). The levels of interferon‐γ (IFN‐γ) (ML057856) and tumor necrosis factor‐α (TNF‐α) (ML002953) in the supernatant were detected by ELISA. Commercial test kits from Enzyme‐Linked Biotech Co., Ltd were used for ELISA according to the standard protocol.

### 2.11 Statistical analysis

All data were analyzed and plotted using SPSS21.0 (IBM Corp.) and GraphPad Prism 8.01 (GraphPad Software Inc.) Software. Measurement data were expressed as mean ± standard deviation. The *t*‐test was used for data comparison between the two groups, one‐way analysis of variance was used for data comparison among multiple groups, and Tukey's test was used for the post hoc test. A value of *p* < .05 meant statistical significance.

## 3 RESULTS

### 3.1 KIT was down‐expressed in TC cells

**Figure 1:**
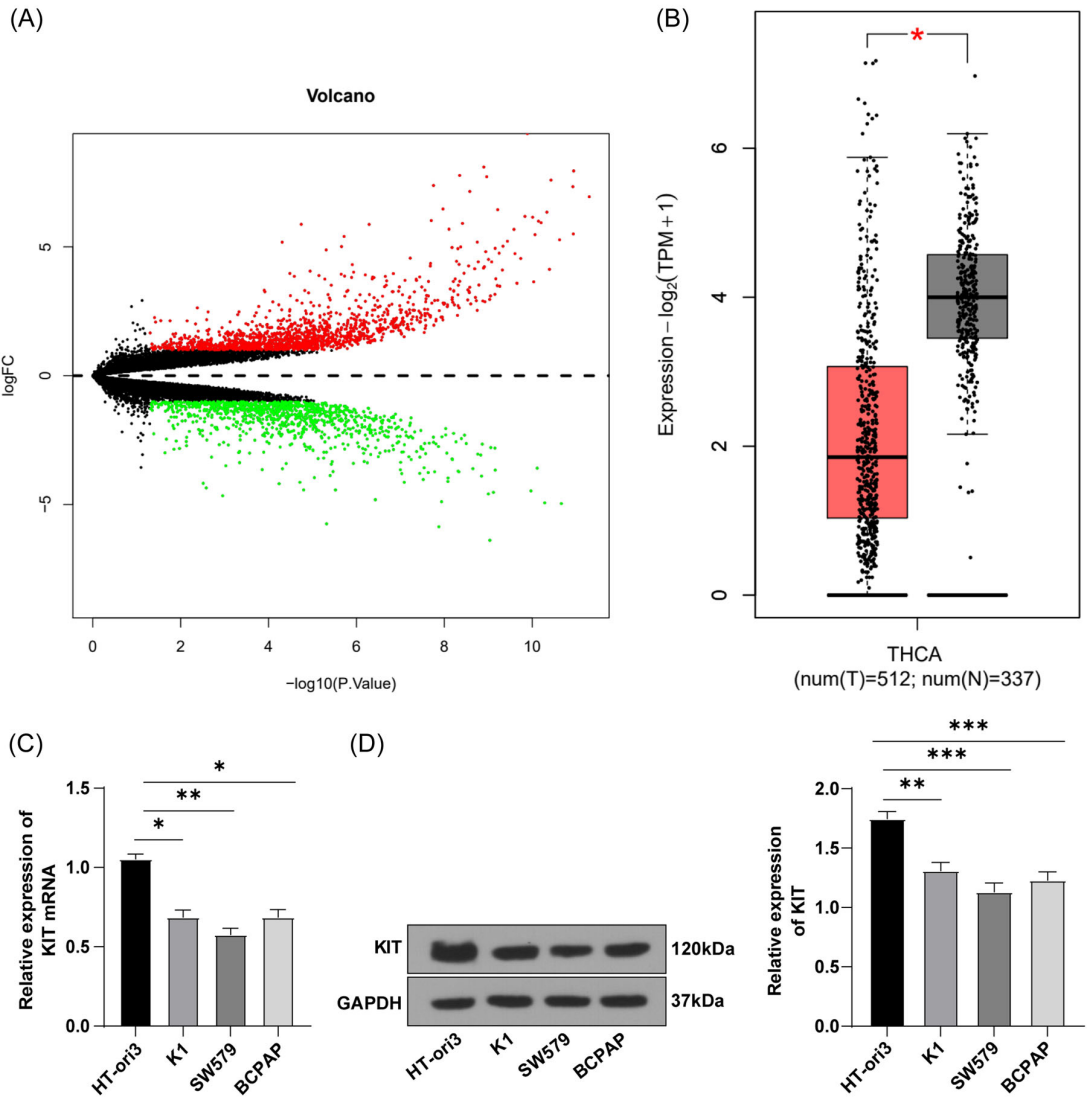
KIT mRNA was lowly expressed in the tissues of TC patients. (A) DEG volcano map of GSE97001 microarray. The abscissa represents logFC and the ordinate represents −log10 *p* value. The red dots represent overexpressed genes and the green dots represent under‐expressed genes. (B) Expression of KIT in TC and normal samples collected by TCGA and GTEx. Red box represents the tumor sample and gray box represents the normal sample. (C) KIT mRNA levels in cells were detected by RT‐qPCR. (D) WB was used to detect KIT protein level in cells. Cell experiment was repeated three times. Values were expressed as mean ± SD. The data comparison between the two groups in (B) was analyzed using *t*‐test. One‐way ANOVA was used for data comparison between multiple groups in (C, D). Tukey's test was used for the post hoc test. *p < .05, **p < .01, ***p < .001. ANOVA, analysis of variance; DEG, differentially expressed genes; GTEx, genotype‐tissue expression; RT‐qPCR, reverse transcription quantitative polymerase chain reaction; SD, standard deviation; TC, thyroid cancer; TCGA, The Cancer Genome Atlas; WB, western blot analysis.

Numerous DEGs have been reported in TC.^23^ To explore the DEGs closely related to TC development, we obtained the TC expression microarray GSE97001 through the GEO database. Next, we conducted differential analysis on gene expression in the microarray and finally identified 2472 DEGs (Figure 1A). In addition, in TC and normal samples collected by TCGA and GTEx, we observed visibly lowered KIT expression in TC (Figure 1B). To further analyze the expression pattern of KIT in TC, we cultured TC cell lines (K1, SW579, and BCPAP) and human normal thyroid cell lines (HT‐ori3) in vitro. The results displayed that the mRNA and protein level of KIT in TC cells was lower than that in normal thyroid cells (all *p* < .01), and KIT expression was the lowest in SW579 cells (Figure 1C,D). These results implied that KIT was lowly‐expressed in TC cells.

### 3.2 Overexpression of KIT inhibited immune escape of TC cells

**Figure 2:**
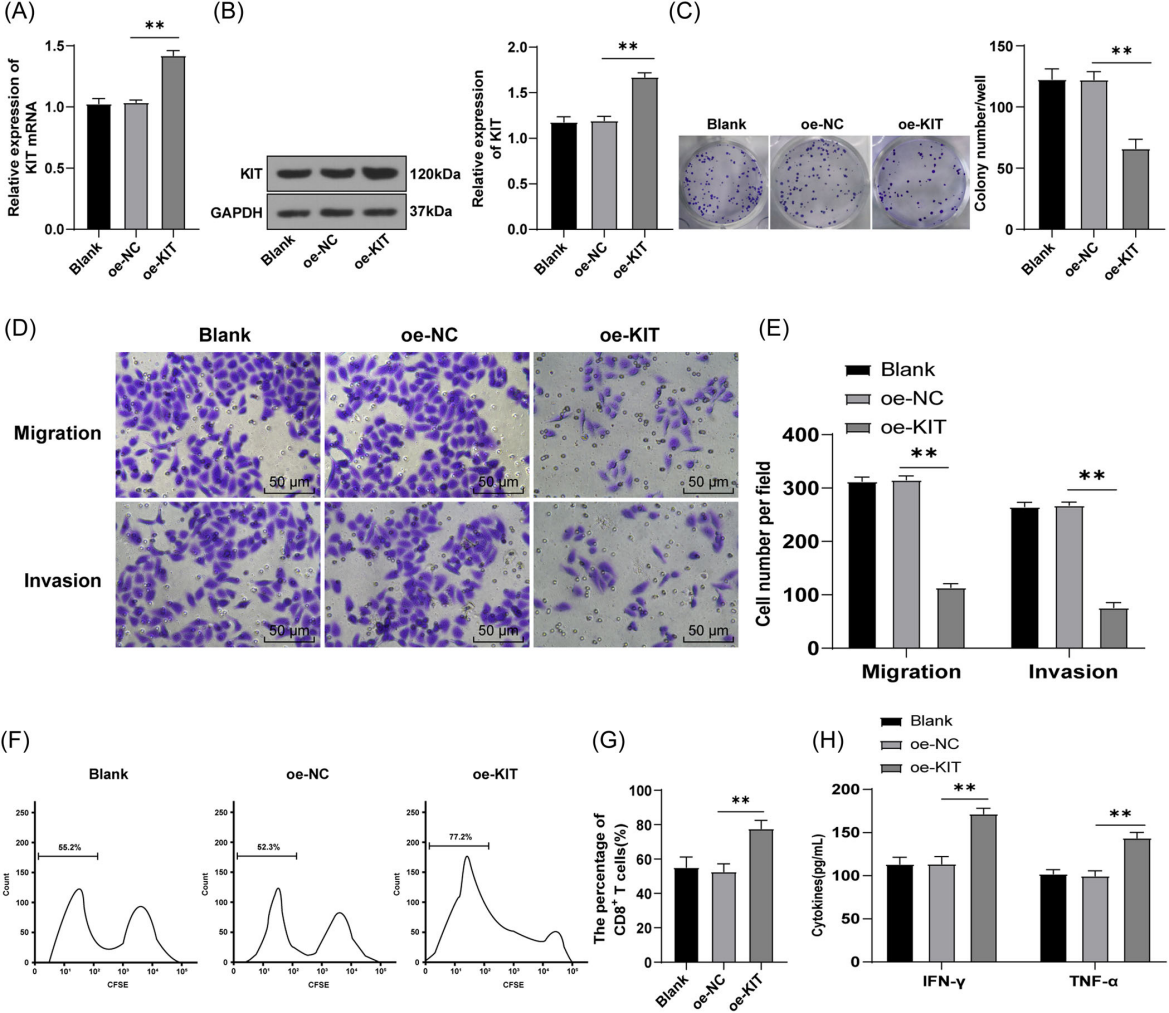
Overexpression of KIT inhibited immune escape of TC cells. (A) RT‐qPCR was used to detect KIT mRNA levels in cells. (B) WB was used to detect the protein level of KIT. (C) The proliferation of SW579 cells was detected by colony formation assay. (D, E) Transwell assays were used to detect the migration and invasion of cells. (F, G) Proliferation of CD8+ T cells labeled with CFSE was detected by flow cytometry. (H) The release of cytokines (IFN‐γ and TNF‐α) was detected by ELISA. Cell experiment was repeated three times. Values were expressed as mean ± SD. One‐way ANOVA was used for data comparison between groups and Tukey's test was used for the post hoc test. **p < .01. ANOVA, analysis of variance; CFSE, carboxyfluorescein succinimidyl ester; ELISA, enzyme‐linked immunosorbent assay; IFN‐γ, interferon‐γ; RT‐qPCR, reverse transcription quantitative polymerase chain reaction; SD, standard deviation; TC, thyroid cancer; TNF‐α, tumor necrosis factor‐α; WB, western blot analysis.

Immune escape is prevalent in the tumor immune cycle.^8^ To explore the impact of KIT on immune escape in TC, SW579 cells with relatively low KIT levels were selected for subsequent experimental studies. SW579 cells were transfected with oe‐KIT to overexpress KIT and then the expression of KIT was detected by RT‐qPCR and WB. The results revealed that the mRNA and protein levels of KIT in the oe‐KIT group were higher than those in the oe‐NC group (*p* < .05) (Figure 2A,B), indicating successful transfection. Subsequently, a colony formation assay was employed to detect the proliferation of SW579 cells, which illustrated that the proliferation ability of cells in the oe‐KIT group was reduced compared with the oe‐NC group (*p* < .01) (Figure 2C). Additionally, Transwell assays showed that the migration and invasion ability of cells in the oe‐KIT group was lower than that in the oe‐NC group (all *p* < .01) (Figure 2D,E). Thereafter, the effects of KIT on T cell proliferation and activation were explored. CD8+ T cells were stained with CFSE and then cocultured with SW579 cells. Flow cytometry was utilized to examine the proliferation of CD8+ T cells, and it was discovered that overexpression of KIT increased the proliferation level of CD8+ T cells (Figure 2F,G). Moreover, ELISA showed that cytokines IFN‐γ and TNF‐α secreted by CD8+ T cells were significantly enhanced (all *p* < .01) (Figure 2H). Taken together, overexpression of KIT can inhibit the immune escape of TC cells.

### 3.3 KIT inhibited the activation of the MAPK pathway in TC cells

**Figure 3:**
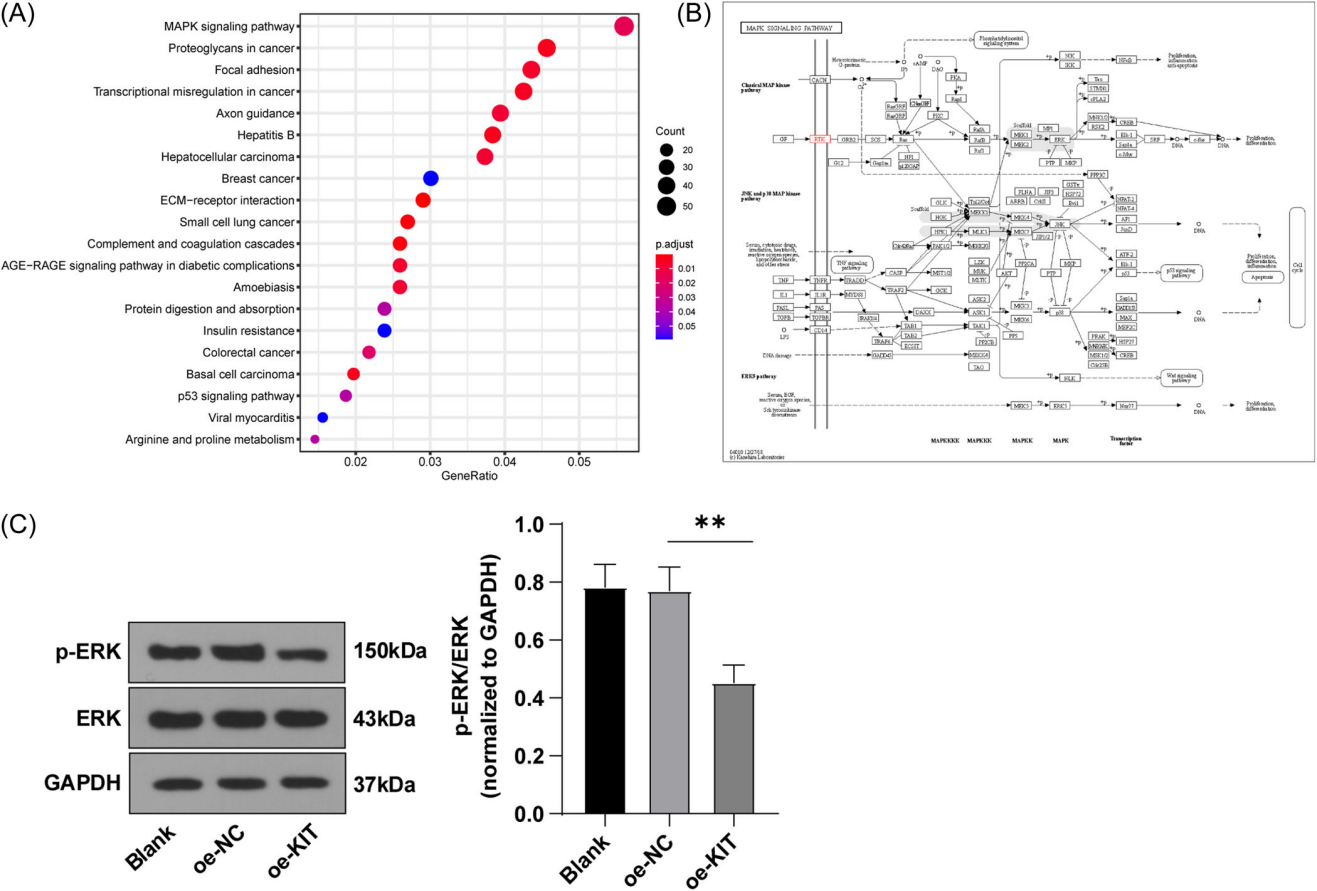
KIT inhibited the activation of the MAPK pathway in TC cells. (A) KEGG pathway enrichment analysis of DEGs. The abscissa represents the GeneRatio and the ordinate represents the name of the gene‐enriched pathway. The circle size and color represent the number of enriched genes and enriched *p* value in the item, and the histogram on the right is color level. (B) Map of the MAPK pathway in KEGG database. Red is the location of the KIT gene in the MAPK pathway. (C) The levels of p‐ERK and ERK in cells were determined by WB. Cell experiment was repeated three times. Values were expressed as mean ± SD. One‐way ANOVA was used for data comparison between groups and Tukey's test was used for the post hoc test. **p < .01. ANOVA, analysis of variance; DEG, differentially expressed genes; KEGG, Kyoto encyclopedia of genes and genomes; SD, standard deviation; TC, thyroid cancer; WB, western blot analysis.

The MAPK pathway is involved in tumor immune escape.^24^, ^25^ KEGG pathway enrichment analysis was performed on DEGs screened from the TC expression microarray GSE97001, which uncovered that these DEGs were mainly enriched in the MAPK pathway (Figure 3A). Among the DEGs enriched in the MAPK pathway, it was observed that KIT was not only located upstream of the MAPK pathway (Figure 3B) but also had the largest differential change multiple in the microarray (Supporting Information: Table 1), indicating that the expression change of KIT may directly affect the activity of the MAPK pathway.

To further ascertain the effects of KIT on the MAPK pathway, we overexpressed KIT in SW579 cells. WB was used to measure the phosphorylation level of the MAPK pathway‐related protein (ERK). The results demonstrated that p‐ERK/ERK levels in the oe‐KIT group were less than those in the oe‐NC group (*p* < .01) (Figure 3C). On the whole, KIT can inhibit the activation of the MAPK pathway in TC cells.

### 3.4 KIT downregulated PD‐L1 expression by regulating the MAPK pathway

**Figure 4:**
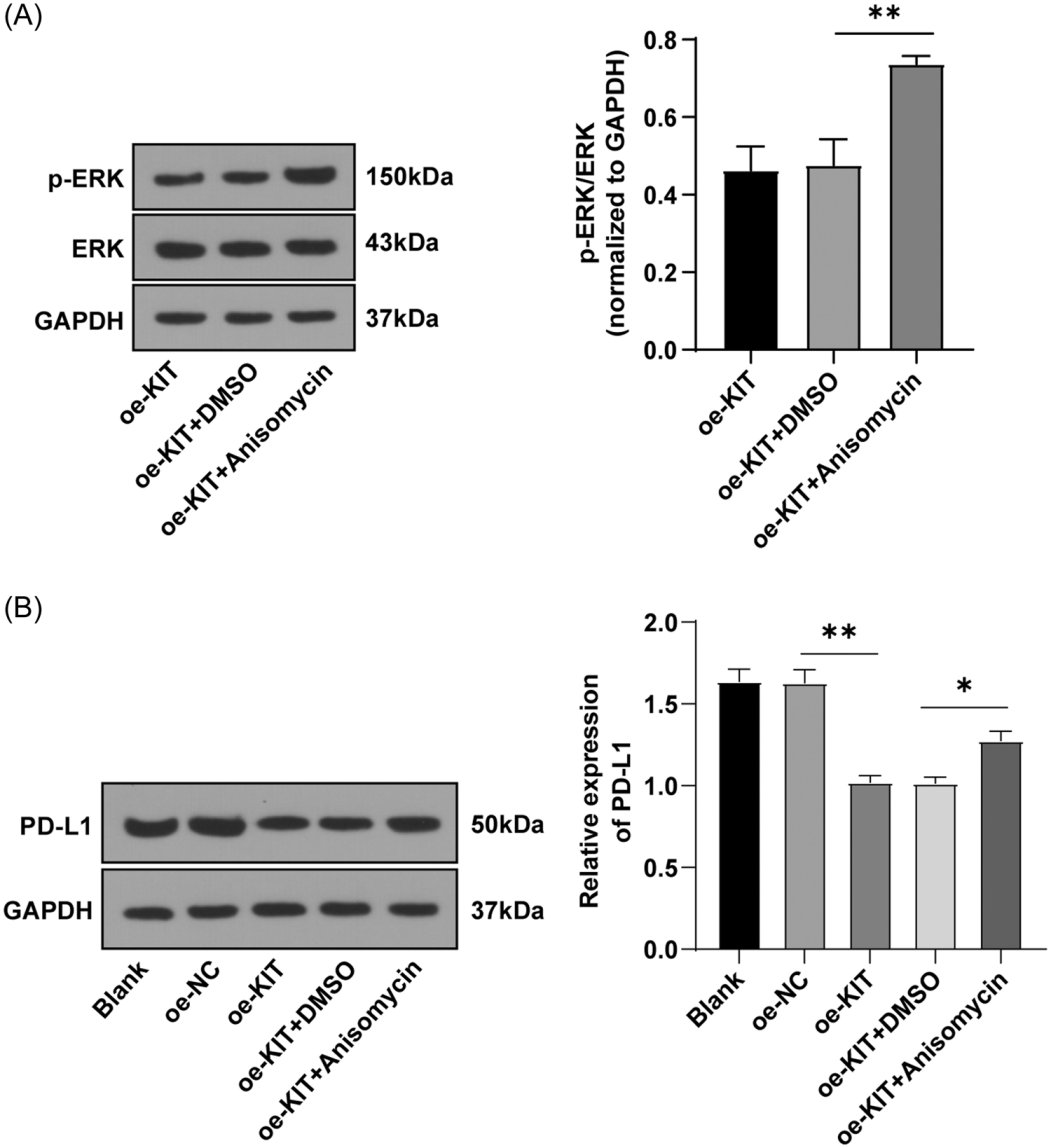
KIT downregulated PD‐L1 expression by regulating the activation of the MAPK pathway. (A, B) The levels of p‐ERK/ERK and PD‐L1 protein were detected by WB. Cell experiment was repeated three times. Values were expressed as mean ± SD. One‐way ANOVA was used for data comparison between groups and Tukey's test was used for the post hoc test. *p < .05, **p < .01. ANOVA, analysis of variance; PD‐L1, programmed death‐ligand 1; SD, standard deviation; WB, western blot analysis.

PD‐L1 is an important immunosuppressive molecule and its high expression in tumor cells promotes tumor immune escape ability.^12^ To further explore whether KIT regulates the PD‐L1 expression through the MAPK pathway, we transfected cells with oe‐KIT and treated cells with anisomycin, an activator of the MAPK pathway. The p‐ERK/ERK and PD‐L1 protein levels were examined by WB. The results showed that p‐ERK/ERK levels were enhanced in the oe‐KIT + Anisomycin group compared with the oe‐KIT + DMSO group (*p* < .01) (Figure 4A); compared with the oe‐NC group, PD‐L1 protein level in the oe‐KIT group was reduced; compared with the oe‐KIT + DMSO group, PD‐L1 protein level in the oe‐KIT + Anisomycin group was raised (all *p* < .05) (Figure 4B). In short, KIT suppressed PD‐L1 level by regulating the activation of the MAPK pathway.

## 4 DISCUSSION

TC is a kind of endocrine system tumor, and its incidence is increasing.^26^, ^27^ To date, a great deal of information has been accumulated about the molecular pathogenesis of PTC, in particular, the activation of the MAPK pathway, which is known to be important in TC.^28^, ^29^ Recently, c‐KIT has been reported to be involved in thyroid cell differentiation and tumor progression.^18^ Nonetheless, the molecular pathogenesis of TC is not thoroughly understood so far.^30^ Our study discovered that KIT suppressed the immune escape of TC by blocking the stimulation of the MAPK pathway and downregulating the expression of PD‐L1.

With the development of RNA sequencing technology and chip technology, the study of DEGs between cancer and normal tissues can improve our understanding of the molecular mechanism of PTC.^31^ RTKs play an important role in many cellular processes, and their dysregulation leads to disease, most importantly cancers.^32^ c‐KIT, a type III RTK, may be involved in thyroid cell differentiation and tumor progression.^18^ In our present study, 2472 DEGs in TC were identified from the GEO database. In addition, by analyzing the expression pattern of KIT in tumors and normal samples included in TCGA and GTEx, we found reduced KIT expression in TC. More importantly, KIT mRNA and protein levels in TC cells were lower than that in normal thyroid cells, which was consistent with the trend of results obtained in public database analysis. Likewise, c‐KIT/CD117, as a new auxiliary marker of PTC, is downregulated in PTC.^17^ In summary, KIT was poorly expressed in TC cells.

T‐cell exhaustion, one of the contributors to tumor immune escape is characterized by reduced levels of TNF‐α and IFN‐γ and impaired CD8+ T cell cytotoxicity.^33^, ^34^ Immune escape is considered to be the phenomenon of tumor cells escaping from the immune system and leading to tumor growth, which is prevalent in the tumor immune cycle.^8^ For example, prostaglandin E2 secreted by TC cells promotes immune escape by inhibiting cytotoxicity of natural killer cells (NK) and NK cell differentiation.^35^ RTKs establish immunosuppression and promote the development of clinically detectable tumors.^36^ Consequently, we hypothesized that abnormal expression of KIT could alter the immune escape of TC cells. Expectedly, after KIT overexpression, the proliferation abilities of TC cells were lowered, as well as the migration and invasion abilities of cells. Consistent with our results, Franceschi et al. revealed in their work that c‐KIT overexpression resulted in significant inhibition of cell proliferation.^18^ CD8+ T cells contribute to tumor eradication and preventing tumors escape from the immune system.^37^ Next, the effects of KIT on proliferation and activation of T cells were investigated by coculture of CFSE‐stained CD8+ T cells and TC cells. Not surprisingly, overexpression of KIT visibly elevated proliferation levels of CD8+ T cells, along with raised levels of cytokines (IFN‐γ/TNF‐α). Promoting the T‐cell proliferation and secretion of cytokines TNF‐α and IFN‐γ can effectively promote the capacity of CD8+ T cells to kill tumor cells and elevate CD8+ T‐cell immune response against tumors.^33^, ^38^ KIT can mediate T cell responses, and the regulation of c‐KIT activity may be the therapeutic target of myeloid‐derived suppressor cells.^39^ Preclinical data suggest that the targeting pathway by the KIT may modulate innate immune cell numbers and activity in tumors.^19^ CD117 may shape the pattern of CD8+ T cell immunodominance during the primary immune response, therefore, CD117 expression may be a potential mechanism of tumor immune evasion.^40^ Collectively, overexpression of KIT repressed immune escape of TC cells.

Accumulated evidence reports that the MAPK pathway is activated in TC and is involved in tumor immune escape.^24^, ^25^, ^41^ Through KEGG pathway enrichment analysis, it was found that DEGs were primarily enriched in the MAPK pathway. Among these DEGs, KIT was not only located upstream of the MAPK pathway but also had the largest differential change multiple in the chip. This suggests that the expression changes of KIT may directly affect the activity of the MAPK pathway. This conclusion is also strongly supported by reports that KIT is involved in signal transduction by regulating different downstream pathways such as MAPK.^20^ We then further overexpressed KIT in TC cells and detected the MAPK pathway‐related p‐ERK/ERK levels were declined in KIT‐overexpressed cells. c‐KIT mutations led to a significant decrease in MAPK activity in adenoid cystic cancer cells.^42^ Tumors from animals were more invasive and had higher RAS/MAPK pathway activation, while KIT knockdown increased RAS/MAPK pathway activation in a BRAF‐mutant human melanoma cell line.^43^ Our findings consistently demonstrated that KIT inhibited the activation of MAPK signaling in TC cells.

As an important immunosuppressive molecule, PD‐L1 is abnormally expressed in tumor cells and is the main factor encouraging tumor immune escape ability.^12^ Additionally, the expression of PD‐L1 was related to the MAPK pathway.^44^ Hence, we overexpressed KIT and simultaneously activated the MAPK pathway using its activator anisomycin. It turned out that PD‐L1 protein levels were diminished in KIT overexpressed cells, while PD‐L1 protein levels were enhanced in cells with an activated MAPK pathway. The downregulation of the MAPK pathway reduces PD‐L1 levels and promotes the proliferation and activation of T cells, while the increase of PD‐L1 expression stimulates immune escape.^24^ Altogether, KIT downregulated PD‐L1 expression by blocking the activation of the MAPK pathway.

In conclusion, our study illustrated that KIT downregulated PD‐L1 levels by inactivating the MAPK pathway, thus inhibiting the immune escape of TC, which provides a new target for the treatment of TC. However, this study only preliminarily considered the mechanism of KIT‐mediated immune escape in TC and did not conduct in‐depth discussions on the upstream regulation of KIT. In addition, only cell experiments were carried out, without in vivo experiments, to verify the role of KIT‐mediated TC. In future studies, we need to further analyze the upstream miRNAs of KIT and perform in vivo experiments to verify the role and mechanism of KIT‐mediated immune escape in TC.

## AUTHOR CONTRIBUTIONS

Zhen Luo guarantor of integrity of the entire study. Rongjun Zhou is responsible for research concepts and study design. Zhen Luo and Dayong Xu are responsible for literature research and experimental studies. Jiaojiao Xu is responsible for data collection and manuscript preparation. Jin Xu and Dayong Xu are responsible for manuscript editing. Keping Deng is responsible for the definition of knowledge content and statistical analysis. Zheng Chen is responsible for manuscript review. Fang Zou is responsible for data analysis. Libo Yao and Yuqin Hu are responsible for clinical research. All authors read and approved the final manuscript.

## CONFLICT OF INTEREST STATEMENT

The authors declare no conflict of interest.

## ETHICS STATEMENT

This article does not contain any studies with human participants or animals performed by any of the authors.

## Supporting information

Supplementary Table 1

## Data Availability

The data that support the findings of this study are available from the corresponding author upon reasonable request.
